# Cannabinoid CB_1_ Receptors Are Localized in Striated Muscle Mitochondria and Regulate Mitochondrial Respiration

**DOI:** 10.3389/fphys.2016.00476

**Published:** 2016-10-25

**Authors:** Juan Mendizabal-Zubiaga, Su Melser, Giovanni Bénard, Almudena Ramos, Leire Reguero, Sergio Arrabal, Izaskun Elezgarai, Inmaculada Gerrikagoitia, Juan Suarez, Fernando Rodríguez De Fonseca, Nagore Puente, Giovanni Marsicano, Pedro Grandes

**Affiliations:** ^1^Department of Neurosciences, Faculty of Medicine and Nursing, University of the Basque CountryLeioa, Spain; ^2^Achucarro Basque Center for Neuroscience, Bizkaia Science and Technology ParkZamudio, Spain; ^3^Group “Endocannabinoids and Neuroadaptation,” NeuroCentre Magendie, Institut National de La Santé et de La Recherche Médicale, U81215Bordeaux, France; ^4^Group “Endocannabinoids and Neuroadaptation,” NeuroCentre Magendie, Université de BordeauxBordeaux, France; ^5^Unidad de Gestión Clínica de Salud Mental, Instituto de Investigación Biomédica de Málaga, Hospital Regional Universitario de MálagaMálaga, Spain; ^6^Division of Medical Sciences, University of VictoriaVictoria, BC, Canada

**Keywords:** endocannabinoid system, intracellular receptors, striated muscle, mitochondrial respiration, metabolism, immunocytochemistry

## Abstract

The cannabinoid type 1 (CB_1_) receptor is widely distributed in the brain and peripheral organs where it regulates cellular functions and metabolism. In the brain, CB_1_ is mainly localized on presynaptic axon terminals but is also found on mitochondria (mtCB_1_), where it regulates cellular respiration and energy production. Likewise, CB_1_ is localized on muscle mitochondria, but very little is known about it. The aim of this study was to further investigate in detail the distribution and functional role of mtCB_1_ in three different striated muscles. Immunoelectron microscopy for CB_1_ was used in skeletal muscles (gastrocnemius and rectus abdominis) and myocardium from wild-type and *CB*_*1*_-KO mice. Functional assessments were performed in mitochondria purified from the heart of the mice and the mitochondrial oxygen consumption upon application of different acute delta-9-tetrahydrocannabinol (Δ^9^-THC) concentrations (100 nM or 200 nM) was monitored. About 26% of the mitochondrial profiles in gastrocnemius, 22% in the rectus abdominis and 17% in the myocardium expressed CB_1_. Furthermore, the proportion of mtCB1 versus total CB_1_ immunoparticles was about 60% in the gastrocnemius, 55% in the rectus abdominis and 78% in the myocardium. Importantly, the CB_1_ immunolabeling pattern disappeared in muscles of *CB*_*1*_-KO mice. Functionally, acute 100 nM or 200 nM THC treatment specifically decreased mitochondria coupled respiration between 12 and 15% in wild-type isolated mitochondria of myocardial muscles but no significant difference was noticed between THC treated and vehicle in mitochondria isolated from *CB*_*1*_-KO heart. Furthermore, gene expression of key enzymes involved in pyruvate synthesis, tricarboxylic acid (TCA) cycle and mitochondrial respiratory chain was evaluated in the striated muscle of *CB*_*1*_-WT and *CB*_*1*_-KO. *CB*_*1*_-KO showed an increase in the gene expression of *Eno3, Pkm2*, and *Pdha1*, suggesting an increased production of pyruvate. In contrast, no significant difference was observed in the *Sdha* and *Cox4i1* expression, between *CB*_*1*_-WT and *CB*_*1*_-KO. In conclusion, CB_1_ receptors in skeletal and myocardial muscles are predominantly localized in mitochondria. The activation of mtCB_1_ receptors may participate in the mitochondrial regulation of the oxidative activity probably through the relevant enzymes implicated in the pyruvate metabolism, a main substrate for TCA activity.

## Introduction

The endocannabinoid system is broadly distributed in the central nervous system (Freund et al., 2003; Castillo et al., 2012; Kano, 2014). This modulatory system is made up of the endocannabinoids (the two main are N-arachidonoylethanolamine or anandamide and 2-arachidonoylglycerol, 2-AG) (Mechoulam et al., 1995; Sugiura et al., 1995) as well as their biosynthetic and degrading enzymes (Freund et al., 2003; Di Marzo et al., 2015) and transport systems (Fu et al., 2011). Most of the cannabinoid and endocannabinoid effects are mediated by the G protein-coupled CB_1_ and CB_2_ receptors (Di Marzo et al., 2015). CB_1_ receptors are also distributed in the periphery, e.g., skeletal muscle, liver, pancreas, and adipose tissue, where they are involved in cellular functions and energy metabolism (Piomelli, 2003; Pagotto et al., 2006; Silvestri and Di Marzo, 2013; Mazier et al., 2015). Anatomically, the protein and mRNA expression of CB_1_ and other components of the endocannabinoid system were demonstrated in human and rodent muscular tissue (Cavuoto et al., 2007b; Esposito et al., 2008; Eckardt et al., 2009; Lipina et al., 2010; Crespillo et al., 2011), in particular in the primary myotubes of skeletal muscle and the rectus abdominis as well as in cultured rat muscular cells (Lipina et al., 2010). Functionally, CB_1_ signaling in skeletal muscle has a negative effect on the expression of genes regulating oxidation and insulin sensitivity (Cavuoto et al., 2007a; Hardie, 2011), modulates the glucose/pyruvate/lactate pathways, regulates mitochondrial dihydrolipoamide dehydrogenase (Arrabal et al., 2015) and controls myoblast differentiation (Iannotti et al., 2014). Therefore, the function and dysfunction of the endocannabinoid system in muscle is a great focus of research interest in order to better understand the underlying mechanisms of metabolic disorders.

In the central nervous system (CNS), CB_1_ receptors are localized on the plasma membrane of synaptic terminals, but also in intracellular compartments (Rozenfeld and Devi, 2008; Bénard et al., 2012; Hebert-Chatelain et al., 2014a,b). The anatomical localization and functional implication of CB_1_ receptors on mitochondrial membranes (mtCB_1_) of brain cells were demonstrated in our own (Bénard et al., 2012; Hebert-Chatelain et al., 2014a,b) and in other laboratories (Koch et al., 2015; Ma et al., 2015; see also Morozov et al., 2013, 2014; Hebert-Chatelain et al., 2014a; Piomelli, 2014 for methodological discussions). Thus, activation of brain mtCB_1_ receptors reduces mitochondrial respiration and ATP production (Bénard et al., 2012; Hebert-Chatelain et al., 2014a,b). In the hypothalamus, activation of CB_1_ receptors negatively regulates leptin-induced reactive oxygen species formation (Palomba et al., 2015) and increases coupled mitochondria respiration that associates with the generation of reactive oxygen species (Koch et al., 2015). CB_1_ has also been shown in our laboratories to be present in muscle mitochondria where it regulates mitochondrial oxidative activity (Arrabal et al., 2015). However, a detailed anatomical and functional characterization of mtCB_1_ in striated muscles is still pending. The aim of this study was to investigate in detail the distribution and functional role of mtCB_1_ in three different striated muscles.

To investigate this, specific CB_1_ antibodies applied to two skeletal muscles (gastrocnemius and rectus abdominis) and myocardium of wild-type and knock-out mice, were combined with a high resolution pre-embedding immunocytochemical method for electron microscopy, followed by strict quantification. Moreover, the impact of CB_1_ agonism on oxygen consumption (respiration) of muscle purified mitochondria from wild-type and *CB*_*1*_-KO mice was also analyzed.

## Materials and methods

### Animal treatment

The procedures were carried out in accordance with European Communities Council Directives (2003/65/CE and 2010/63/UE) and current Spanish regulations (Real Decreto 53/2013 and Ley 32/2007). The protocols for animal care and use were approved by the appropriate Committee at the University of the Basque Country UPV/EHU (CEBA/93/2010/GRANDESMORENO). Furthermore, great efforts were made in order to minimize the number and suffering of the animals used.

6 wild-type C57BL/6N female mice (3–5 month old) and 3 *CB*_*1*_-KO female mice were used. *CB*_*1*_-KO mice were obtained, bred and genotyped as described (Marsicano et al., 2002). The line was in a mixed genetic background, with a predominant C57BL/6N contribution. Animals were maintained under standard conditions (12 h light/dark cycle) with food (standard chow, Global Diet 2014S, Harlan) and water *ad libitum*. During the light cycle, mice were deeply anesthetized by intraperitoneal injection of ketamine/xilazine (80/10 mg/kg body weight) and were transcardially perfused at room temperature (RT, 20–25°C) with phosphate-buffered saline (PBS 0.1 M, pH 7.4) for 20 s, followed by the fixative solution made up of 4% formaldehyde (freshly depolymerized from paraformaldehyde), 0.2% picric acid and 0.1% glutaraldehyde in a phosphate buffer (PB 0.1 M, pH 7.4) for 10–15 min. Then, muscles (rectus abdominis, gastrocnemius and myocardium) were removed and postfixed in the fixative solution for approximately 1 week at 4°C. Afterwards, they were stored at 4°C in a 1:10 diluted fixative solution until used.

### Preembedding immunogold method for CB_1_ immunoelectron microscopy

Muscular tissues were cut at 50 μm in a vibratome and collected in 0.1 M PB (pH 7.4) at RT. Sections were preincubated in a blocking solution of 10% bovine serum albumin BSA, 0.1% sodium azide and 0.02% saponin prepared in TBS (1X, pH 7.4) for 30 min at RT. A preembedding silver-intensified immunogold method was used for the localization of the CB_1_ protein. Muscular sections were incubated in the primary goat CB_1_ polyclonal antibodies (2 μg/ml; CB1-Go-Af450-1; Frontier Science Co. Ltd; 1-777-12, Shinko-nishi, Ishikari, Hokkaido, Japan) in 10% BSA/TBS containing 0.1% sodium azide and 0.004% saponin on a shaker for 2 days at 4°C.

After several washes in 1% BSA/TBS, tissue sections were incubated in a secondary 1.4 nm gold-labeled rabbit anti-goat IgG (Fab' fragment, 1:100, Nanoprobes Inc., Yaphank, NY, USA) in 1% BSA/TBS with 0.004% saponin on a shaker for 4 h at RT. Thereafter, the tissue was washed in 1% BSA/TBS, stored overnight at 4°C and postfixed in 1% glutaraldehyde in TBS for 10 min at RT. Following the washes in double-distilled water, gold particles were silver-intensified with a HQ Silver kit (Nanoprobes Inc., Yaphank, NY, USA) for about 12 min in the dark and then washed in 0.1 M PB (pH 7.4). Stained sections were osmicated (1% OsO_4_ in 0.1 M PB, pH 7.4, 20 min), dehydrated in graded alcohols to propylene oxide and plastic-embedded flat in Epon 812. 65 nm ultrathin sections were collected on mesh nickel grids, stained with uranyl acetate and lead citrate, and examined in a Philips EM208S electron microscope. Tissue preparations were photographed by using a digital camera coupled to the electron microscope.

#### Semi-quantification of mitochondrial CB_1_ in muscle

Fifty micrometer-thick muscular sections from each animal genotype (*n* = 3 each) were cut at 65 nm and random electron micrographs (10,000–25,000X) were taken from the grids (132 μm side). To avoid false negatives, only ultrathin sections in the first 1.5 μm from the surface of the tissue block were examined. Positive labeling was considered if at least one immunogold particle was over mitochondria or within approximately 30 nm from the mitochondria membrane. Metal particles on mitochondrial membranes were visualized and counted by unbiased observers. The numbers of labeled mitochondria were normalized to the total number of mitochondria in the images in order to establish the proportion of CB_1_-positive mitochondria. Normalized mtCB_1_ labeling versus total CB_1_ defined the proportion of mtCB_1_ versus total CB_1_ as performed previously (Bénard et al., 2012; Hebert-Chatelain et al., 2014a). Density of mitochondrial CB_1_ immunolabeling was calculated as immunoparticles/μm membrane of positive mitochondria. Image-J software (1.43u version, NIH, USA) was used to measure the membrane length and analyzed area. Values within wild-type or *CB*_*1*_-KO littermates did not show any significant difference, allowing the pooling of respective wild-type and *CB*_*1*_-KO littermates.

### Mitochondrial isolation from heart and mitochondrial respiration

Mitochondria were isolated from the heart of the mice by differential centrifugation. Hearts were collected in isolation medium I (in mM: 210 mannitol, 70 sucrose, 50 Tris-HCl, pH 7.4, and 10 K-EDTA), finely cut and then digested by trypsin (0.5 mg/g of heart muscle) for 30 min. The proteolysis was stopped by addition of trypsin inhibitor (soybean 3:1 inhibitor to trypsin). Tissues were homogenized using a tissue homogenizer. The homogenate was centrifuged at 1000 g for 5 min. The supernatant was strained through gauze and centrifuged at 7000 g for 10 min. The resulting pellet was resuspended in ice-cold isolation medium II (in mM: 225 mannitol, 75 sucrose, 10 Tris-HCl, pH 7.4, and 0.1 K-EDTA), and a new series of centrifugations (1000 and 7000 g) was performed. The last mitochondrial pellet was resuspended into a minimum volume of isolation medium II to obtain a mitochondrial concentration between 10 and 40 mg/ml.

Mitochondrial oxygen consumption ratio (OCR) was monitored at 37°C. Isolated mitochondria were placed in an oxygraph chamber equipped with a Clark oxygen electrode (Hansatech) containing 1 ml of respiration buffer (75 mM mannitol, 25 mM sucrose, 100 mM KCl, 10 mM Tris phosphate, 10 mM Tris-HCl, pH 7.4, 5 mM EDTA). The final mitochondrial concentration in the chamber was 1 mg/ml. The respiratory states were obtained by adding consecutively to the chamber Pyruvate and Malate at 10 mM final (state 4) and then, ADP at 2 mM (state 3). THC was added directly into the respiratory chamber (final concentration 100 and 200 nM) during state 3. Oxygen concentration in the chamber was recorded over the time and effect of THC on the OCR was measured between 5 and 10 min after the THC addition. Respiration rates were normalized to protein concentration. The respiratory coupling ratio (RCR) was defined as the ratio of state 3 over state 4.

### Immunoprecipitation

Immunoprecipitation of CB_1_ was performed on the mitochondrial fraction isolated as described above by using the Pierce™ Co-Immunoprecipitation Kit, as per manufacturer's instructions. Briefly, the mitochondrial fraction (625 μg of protein) were diluted in IP/lysis buffer and pre-cleared using a control agarose resin to minimize non-specific binding. These lysates were then applied to columns containing 8 μg immobilized anti-CB_1_ (Abcam, ab23703) covalently linked to an amine-active resin and incubated overnight at 4°C. The immunoprecipitate was then eluted, mixed with Laemmli buffer, heated to 37°C for 30 min and analyzed by SDS-PAGE using conventional methods.

### RNA isolation and qRT-PCR analysis

Total RNA was extracted from striated (rectus abdominis) mouse muscle (~100 mg) by using the Trizol method, as previously described (Arrabal et al., 2015). Purified RNA (1 μg) and random hexamers were used to generate first strand cDNA using transcriptor reverse transcriptase. cDNA was used as a template for quantitative real-time PCR. The relative quantification was normalized to the expression of the housekeeping gene *Gapdh* and calculated by using the ΔΔCt method. Primers used for the qRT-PCR reaction were obtained based on TaqMan® Gene Expression Assays (Life Technologies) (Table 1).

**Table 1:** **Primer references for TaqMan® gene expression assays (ThermoFisher)**.

**Gene name**	**Assay ID**	**Amplicon length**
*Actb*	Mm00607939_s1	115
*Gapdh*	Mm99999915_g1	109
*Gusb*	Mm01197698_m1	71
*Eno3*	Mm00468267_m1	54
*Pkm2*	Mm00834102_gH	182
*Pdha1*	Mm00468675_m1	74
*Sdha*	Mm01352366_m1	82
*Cox4i1*	Mm01250094_m1	116

Actb, β-actin; Cox4i1, cytochrome c oxidase subunit 4 isoform 1, mitocondrial; Eno3, enolase 3, β-muscle; Gapdh, glyceraldehyde 3-phosphate dehydrogenase; Gusb, β-glucuronidase; Pdha1, pyruvate dehydrogenase E1 alpha 1; Pkm2, pyruvate kinase, muscle isozyme; Sdha, succinate dehydrogenase complex, subunit A, flavoprotein variant.

### Statistical analysis

Results were expressed as mean ± S.E.M. of 3–6 determinations per experimental group. For semi-quantification of mitochondrial CB_1_, group differences were compared by the chi-square test. For density of mitochondrial CB_1_ immunolabeling, group differences were analyzed by the Mann Whitney test. For gene expression, group differences were analyzed by one-tailed Student's *t*-test with the Welch correction. A *P*-value below 0.05 was considered statistically significant. Graphs and statistical analyses were performed using GraphPad software 4.0 (GraphPad Software Inc., San Diego, USA). Oxygen consumption measurements: statistics were performed using GraphPad Prism6. Values are means ± S.E.M. Paired *t*-test was used when comparing sequential measurements. One-way ANOVA followed by Sidak multiple comparison test were applied when comparing different groups.

## Results

### Immunolocalization of mitochondrial CB_1_ in muscle

**Figure 1:**
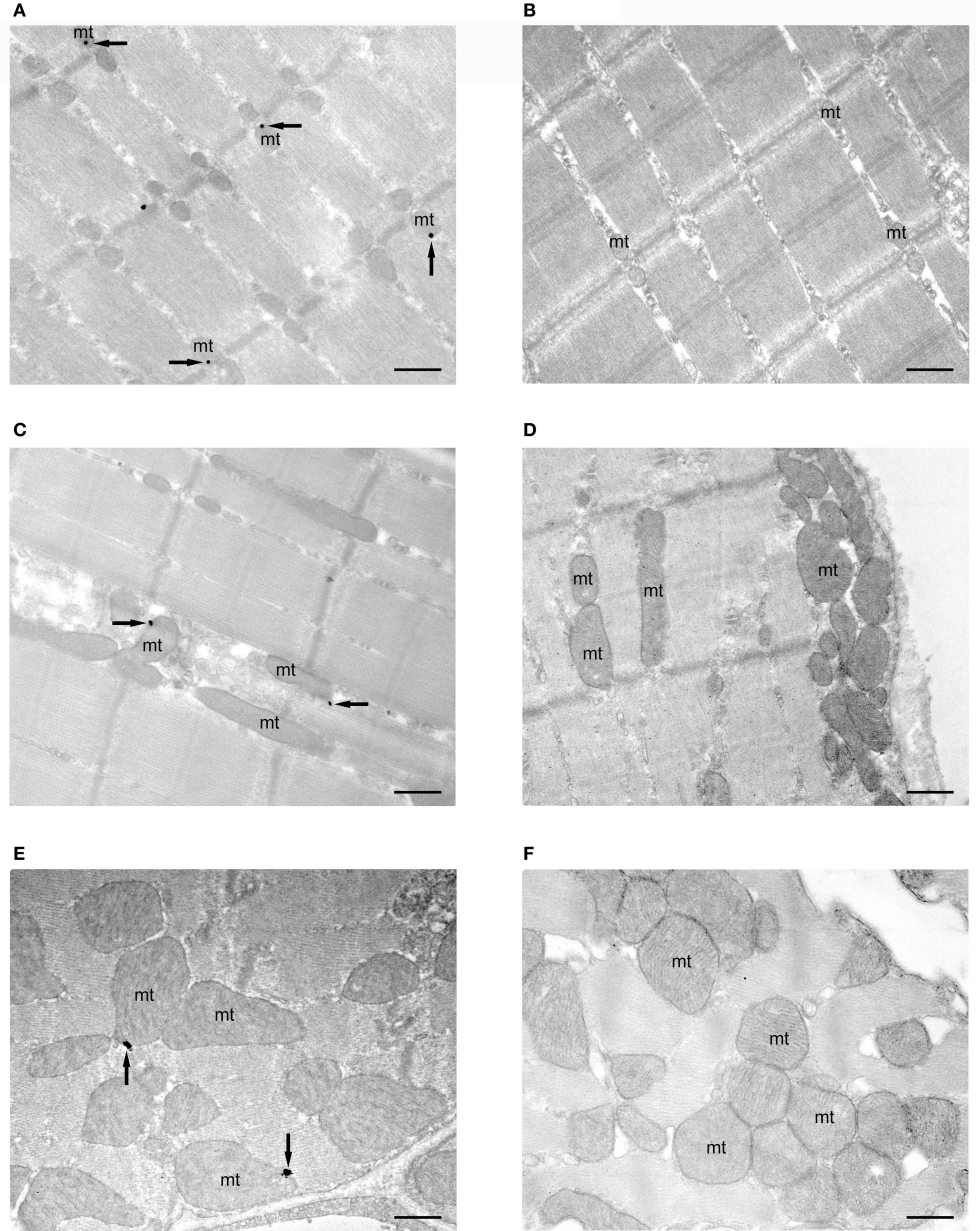
**subcellular localization of CB_1_ receptors in mitochondria of striated muscles**. Preembedding silver-intensified immunogold method for electron microscopy. CB_1_ immunoparticles (arrows) are localized in outer mitochondrial (mt) membranes of the gastrocnemius **(A)**, rectus abdominis **(C)** and myocardial **(E)** muscles of *CB*_*1*_-WT mice. No labeling is observed in mitochondria (mt) of *CB*_*1*_-KO muscles **(B,D,F)**. Scale bars: 0.5 μm. All electron micrographs were taken at the same magnification (x 22,000).

**Figure 2:**
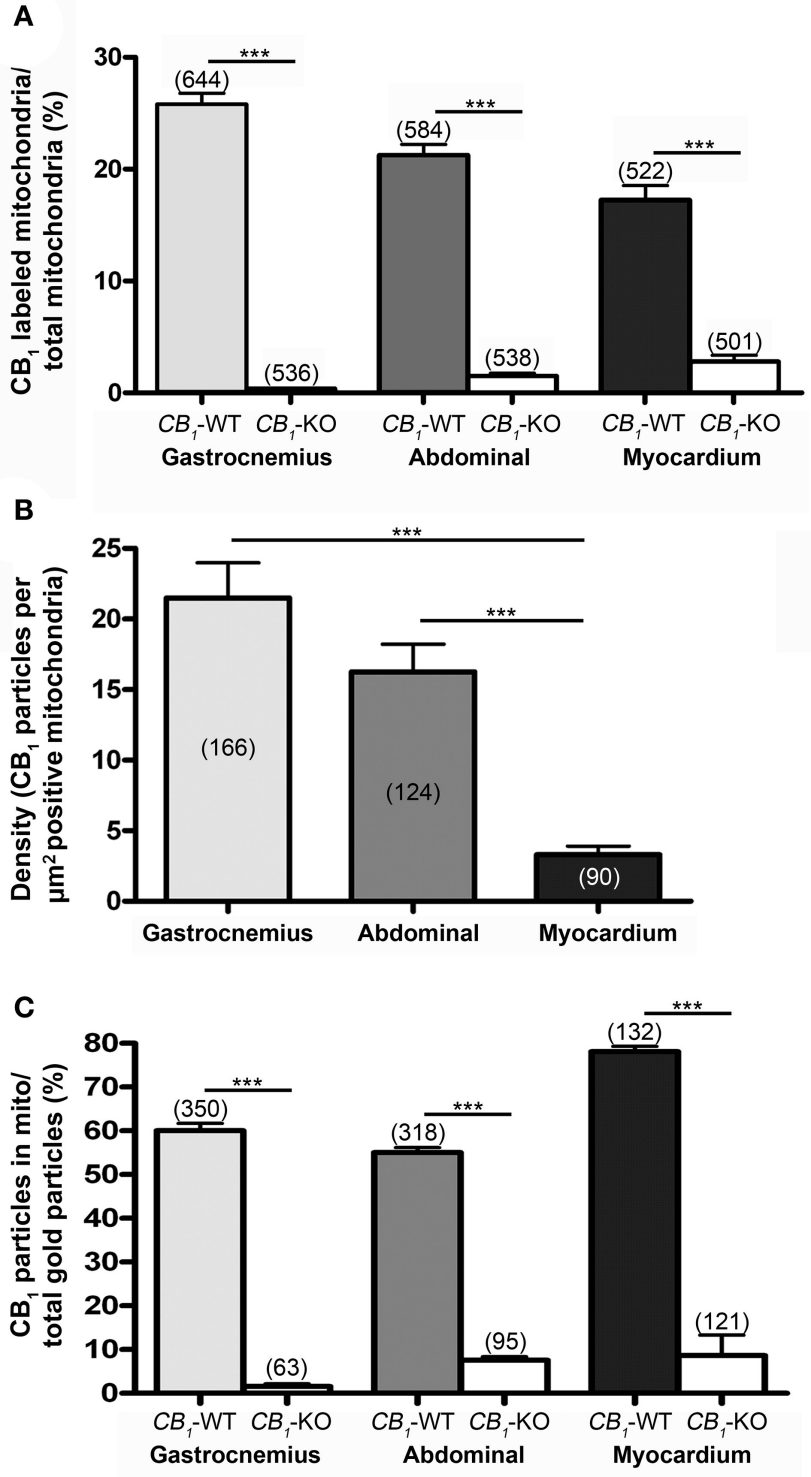
**proportion of CB_1_ immunopositive mitochondria. (A)** Values obtained in the gastrocnemius (25.77 ± 1.02%), rectus abdominis (21.29 ± 0.98%) and myocardium (17.24 ± 1.29%) of 3 different mice. These values are very low in *CB*_*1*_-KO muscles (gastrocnemius: 0.37 ± 0.04%; rectus abdominis: 1.45 ± 0.29%; myocardium: 2.79 ± 0.57%). Numbers between parentheses indicate the number of analyzed profiles. Values mean ± S.E.M. Chi-square test. ^***^P < 0.001 as compared to *CB*_*1*_-WT. **(B)** Density of CB_1_ gold particles per area (μm^2^) of the immunopositive mitochondria in gastrocnemius (21.49 ± 2.51%), rectus abdominis (16.25 ± 1.95%) and myocardium (3.30 ± 0.60%) of *CB*_*1*_-WT. ^***^P < 0.001. Numbers between parentheses indicate the number of analyzed profiles. Density was negligible in *CB*_*1*_-KO, therefore, it was not taken into account to avoid distorting bias. **(C)** Proportion of CB_1_ immunoparticles in mitochondria of the total CB_1_ labeling in gastrocnemius (60 ± 1.73%), rectus abdominis (55.04 ± 1.15%) and myocardium (78.11 ± 1.16%) drops drastically in *CB*_*1*_-KO muscles.

The subcellular compartmentalization of CB_1_ in three different striated muscle tissues of mice by using a preembedding immunogold method for electron microscopy was studied. The gastrocnemius had CB_1_ immunoparticles along the sarcolemma of the muscle fibers. Only scattered immunoparticles were observed at the limits of the sarcomer as well as in the sarcoplasmic reticulum. However, the greatest accumulation of immunoparticles was in the mitochondria (Figure 1). About 26% of the mitochondria in the gastrocnemius were CB_1_ immunopositive (25.77% ± 1.022, *n* = 644) with the majority of gold particles disposed on the outer mitochondrial membrane (Figures 1A, 2A). Importantly, CB_*1*_ staining was virtually absent in the gastrocnemius of mice lacking CB_1_ (*CB*_1_-KO, Figure 1B), since only a few mitochondria (0.37% ± 0.040, *n* = 536) exhibited occasional unspecific background particles (Figure 2A).

A similar distribution pattern of CB_1_ immunostaining was observed in the other muscles studied. Thus, in the rectus abdominis (Figures 1C,D), the proportion of CB_1_ immunopositive mitochondria was slightly lower than in the gastrocnemius (21.29% ± 0.982, *n* = 584) and the background level was estimated to be below 1.5% in the *CB*_*1*_-KO (1.45% ± 0.289, *n* = 538) (Figure 2A). Also, the CB_1_ immunolocalization in the myocardium (Figures 1E,F) was predominantly in mitochondria (17.24% ± 1.293, *n* = 522) with a background of ~2.8% in the *CB*_*1*_-KO (2.79% ± 0.571, *n* = 501) (Figure 2A). Taken together, the mtCB_1_ labeling seems to be highly specific in the three muscles because the percentage of mitochondria with residual staining was negligible in the same muscles of *CB*_*1*_-KO mice processed simultaneously with *CB*_*1*_-WT muscle tissues. Furthermore, the density of CB_1_ immunolabeling, i.e., the number of gold particles per square micron of labeled mitochondrial membrane, was 21.49 ± 2.51 (*n* = 166) in the gastrocnemius, 16.25 ± 1.95 (*n* = 124) in the rectus abdominis and 3.30 ± 0.60 (*n* = 90) in the myocardium (Figure 2B). Lastly, 60 ± 1.7% of the gastrocnemius, 55 ± 1.16% of the rectus abdominis and 78 ± 1.16% of the myocardium total CB_1_ immunoparticles were localized to mitochondria and almost null in *CB*_*1*_-KO (Figure 2C), indicating that the predominant distribution of CB_1_ in the analyzed muscles is in this organelle.

### Functional assays of mitochondrial CB_1_ in muscle

**Figure 3:**
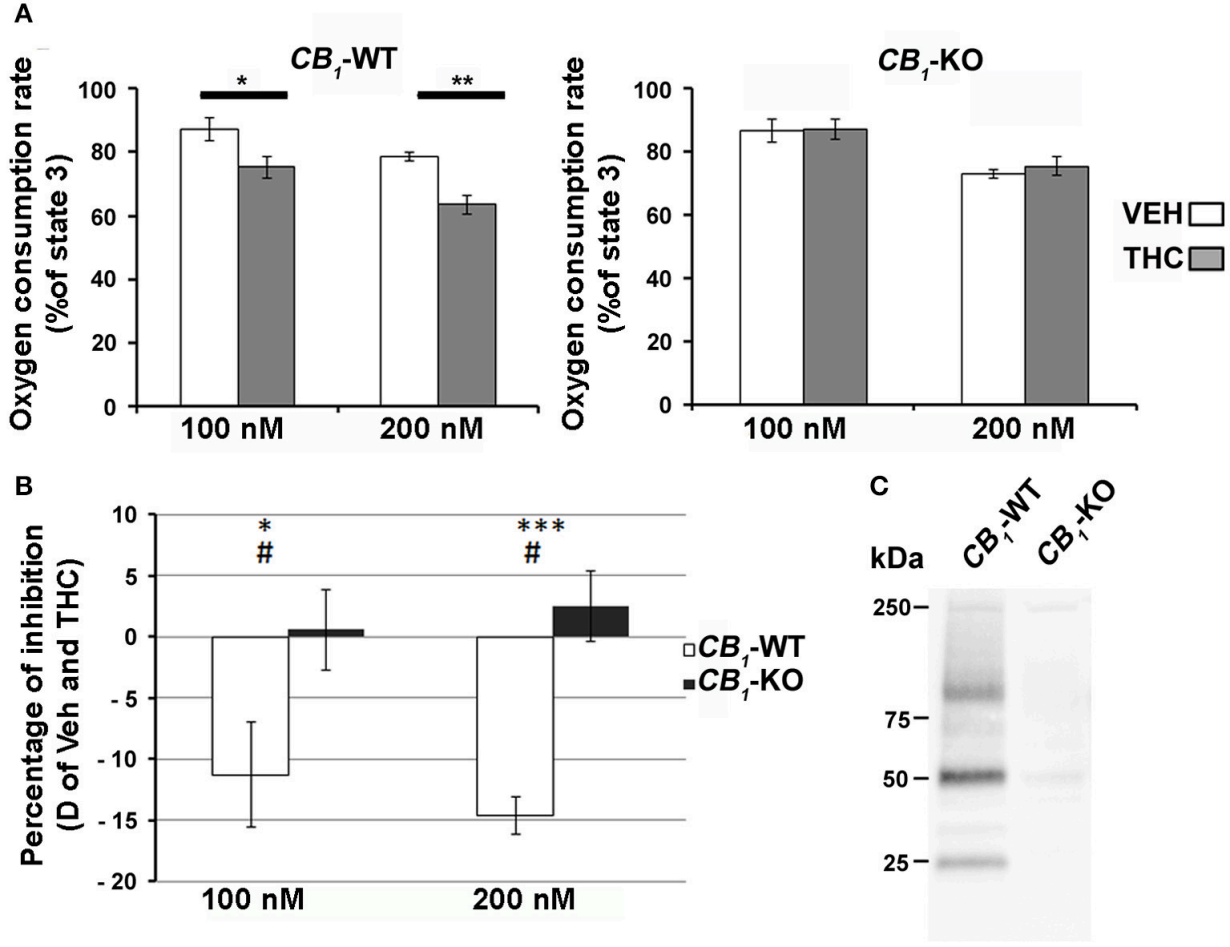
**(A)** Effect of THC on heart isolated mitochondria. THC (100 nM or 200 nM, gray bars) or vehicle (white bars) was added directly to the respiratory chamber during the ADP-dependent respiration. Values are expressed as mean of percentage of the ADP-dependent respiration of *CB*_*1*_-WT (left panel) or *CB*_*1*_-KO (right panel) isolated mitochondria (*n* = 4). **(B)** Difference of inhibition between vehicle and THC dependent respiration (White bars; *CB*_*1*_-WT, Black bars *CB*_*1*_-KO). ^*^P < 0.05 THC vs. VEH; ^**^P < 0.01 THC vs. VEH; ^***^P < 0.001 THC vs. VEH; #*P* < 0.05 *CB*_*1*_-WT vs. *CB*_*1*_-KO. C) Immunoprecipitation of CB_1_ from heart mitochondrial fractions of *CB*_*1*_-WT or *CB*_*1*_-KO.

The regulation of mitochondrial bioenergetic functions by CB_1_ receptors was also investigated. Mitochondrial respiration was measured on heart mitochondria isolated from both wild-type and *CB*_*1*_-KO mice. The focus was on the myocardium because this muscle had the highest proportion of CB_1_ in the mitochondria. There was no difference between KO and wild-type isolated mitochondria regarding basal and coupled respiration (respectively State 4 and State 3; see Table 2) and no difference was found regarding the respiratory coupling ratio (State3/State4). Together, these results showed that both wild-type and KO isolated mitochondria had the same capacity to produce ATP. Yet, acute THC treatment specifically inhibited mitochondrial oxygen consumption in wild-type isolated mitochondria. Adding 100 nM or 200 nM THC respectively decreased the mitochondria coupled respiration by 11.9 ± 3.7% and 15.2 ± 1.3% compared to the vehicle (*n* = 4) (Figure 3A left graph, *CB*_*1*_-WT; Figure 3B). In contrast, no significant difference was noticed between THC treated and vehicle in mitochondria isolated from *CB*_*1*_-KO heart (Figure 3A right graph, *CB*_*1*_-KO; Figure 3B). As a control, immunoprecipitation was performed to test for the presence of CB_1_ in the mitochondrial fractions. The protein extracts from *CB*_*1*_-WT and *CB*_*1*_-KO heart mitochondria fractions were subjected to immunoprecipitation with an antibody against CB_*1*_, and subsequently analyzed through Western blotting. Representative results demonstrate that CB_1_ was precipitated from the *CB*_*1*_-WT mitochondrial fraction but not from the *CB*_*1*_-KO fraction (Figure 3C).

**Table 2:** **Basal (State 4) and coupled (State 3) respiration in isolated Mitochondria**.

**OCR (nMol Oxygen/min/mg prot.)**	***CB_1_*–WT**	***CB_1_*–KO**
Pyruvate+Malate (State 4)	33.54 ± 4.03	38.52 ± 5.06
Pyruvate+Malate+ADP (State 3)	122.08 ± 16.21	132.05 ± 16.24
Coupling ratio	3.75 ± 0.43	3.50 ± 0.26

Absolute value (means ± SEM, n = 6). Differences are non-significative.

### Gene expression of relevant enzymes in pyruvate metabolism, TCA cycle and mitochondrial respiratory chain in the striated muscle

**Figure 4:**
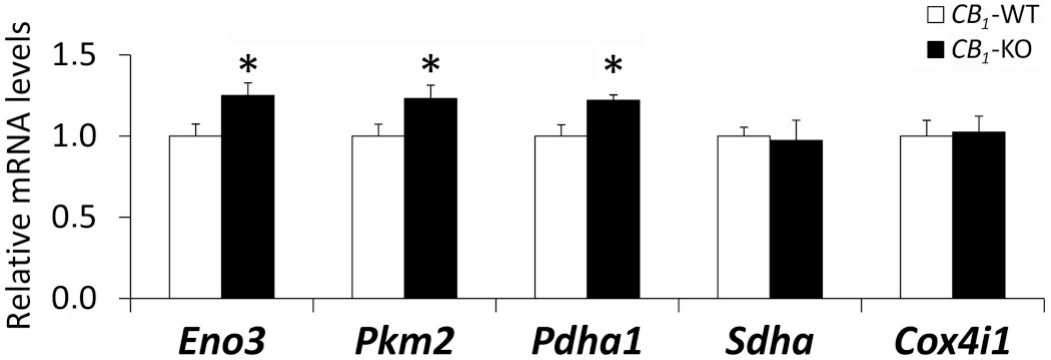
**Gene expression of *Eno3*, *Pkm2*, *Pdha1*, *Sdha* and *Cox4i1* in the striated muscle of *CB*_*1*_-WT and *CB*_*1*_-KO mice**. Student's *t*-test (*n* = 6): ^*^P < 0.05 vs. wild-type.

To further investigate the mitochondrial energetic functions, the gene expression of key enzymes involved in pyruvate synthesis, TCA cycle and mitochondrial respiratory chain in the rectus abdominis of *CB*_*1*_-WT and *CB*_*1*_-KO mice was evaluated (Figure 4). *CB*_*1*_-KO mice showed an increase in the gene expression of *Eno3, Pkm2*, and *Pdha1* (^*^P < 0.05), suggesting an increased production of pyruvate, a main substrate of TCA cycle activity. In contrast, no significant difference was observed in the muscle expression of *Sdha* and *Cox4i1* between *CB*_*1*_-WT and *CB*_*1*_-KO mice (Figure 4).

## Discussion

Following our previous observations in rat and mouse (Arrabal et al., 2015) these data confirm and further extend the presence of CB_1_ in mitochondria of the mouse striated skeletal (gastrocnemius and rectus abdominis) and myocardial muscles. In particular, CB_1_ immunoparticles were localized on the outer mitochondrial membrane similarly to the mtCB_1_ distribution in the *CB*_*1*_-WT hippocampus, with almost absent mtCB_1_ in *CB*_*1*_-KO tissue (Bénard et al., 2012; Hebert-Chatelain et al., 2014a,b). Indeed, about 30% of the mitochondria in axon terminals and somatodendritic domains of neurons contained CB_1_ in the hippocampal CA1 region of *CB*_*1*_-WT, with a background of 3% in *CB*_1_-KO (Bénard et al., 2012). Although mtCB1 labeling has been challenged by using the DAB-Ni technique (Morozov et al., 2013, 2014, 2016) that produces a higher background mitochondrial staining than the immunogold procedure due to the presence of endogenous biotinylated proteins (Hollinshead et al., 1997; Bénard et al., 2012; Hebert-Chatelain et al., 2014a,b), careful quantifications revealed a significantly higher staining of brain mitochondria in wild-type than in *CB*_*1*_-KO brain tissue, regardless of the method used (Bénard et al., 2012; Hebert-Chatelain et al., 2014a,b). Furthermore, with a very restrictive quantification protocol, i.e., taking into account only mitochondrial particles far away from other neuronal membranes, about 22% of the mitochondrial sections were CB_1_ immunopositive in *CB*_*1*_-WT and only 3% exhibited particles in the *CB*_*1*_-KO hippocampal tissue (Hebert-Chatelain et al., 2014a,b).

The amount of mitochondria in striated muscles is more abundant than in the brain because the energy demand in muscle cells and cardiomyocytes is very high. Furthermore, the size, shape and number of mitochondria are constantly changing as a consequence of muscle and heart activity. Therefore, the distribution of CB_1_ receptors may depend on the quantity of muscle mitochondria. The results of this study showed that the proportion of mitochondrial sections equipped with CB_1_ in the muscle was in the range of the brain, indicating that only a certain subpopulation of mitochondria bearing CB_1_ receptors is present in both the CNS and the peripheral striated muscles. Furthermore, the immunolabeling density in the CA1 hippocampus was around 18 particles/μm^2^ and around 15% of the total CB_1_ immunolabeling in CA1 neurons was localized in the mitochondria (Bénard et al., 2012). In muscles, the density was only low in myocardium and the proportion of the total CB_1_ immunoparticles in the mitochondria was between 55 and 78% in the three muscle samples studied, pointing out that despite its low absolute level of expression in the myocardium, but not in the others (Pagotto et al., 2006), the majority of CB_1_ receptors in the muscle is present on mitochondrial membranes. In the brain, the activation of mtCB_1_ receptors induces a reduction of mitochondrial respiration of about 20–30% (Bénard et al., 2012; Hebert-Chatelain et al., 2014a,b). Similarly, THC activation of the CB_1_ receptors in the myocardium mitochondria reduces mitochondrial respiration, although at a lower rate than in the brain. This difference may be explained, among other factors, by the lower amount of CB_1_ receptors (particle density and percentage of positive mitochondria) in the myocardium as compared to the brain, which cannot be compensated by the fact that the vast majority of the total CB_1_ immunoparticles in myocardium are localized in mitochondria.

The two skeletal muscles analyzed, the gastrocnemius and the rectus abdominis, are enriched in type I slow-twitch oxidative fibers (Hijikata et al., 1992). Therefore, a potential role for mtCB_1_ receptors might be the regulation of the oxidative activity at the mitochondria, probably through the relevant enzymes Eno3 and Pkm2, which are implicated in the pyruvate metabolism (a main substrate for TCA cycle), as well as the Pdha and DLD, which are key components of the mitochondrial pyruvate/α-ketoglutarate/branched-chain keto acid (BCKDC) dehydrogenase complexes implicated in the TCA cycle activity (Arrabal et al., 2015). In fact, studies using a CB_1_ receptor antagonist (Rimonabant) demonstrated that a blockade of cannabinoid receptors increases glucose use by striated muscle (Liu et al., 2005; Cavuoto et al., 2007a). Moreover, a previous study indicated that it is mandatory the abetment of a hypercaloric context and a deficiency in CB_1_ receptor activity to observe an increased expression of the mitochondrial respiratory chain component *Cox4i1* and a potential mitochondrial respiration (Arrabal et al., 2015). Together these results indicated an up-regulation of the glucose/pyruvate metabolism in response to increased energy expenditure through mitochondrial oxidation and TCA cycle in the insulin-highly sensitive striated muscle. However, the transcriptional differences in pyruvate metabolism could not reflect changes in the maximal capacity of ATP production, suggesting no evident differences in the basal phenotype of the *CB*_*1*_-KO muscle at functional levels. Indeed, we have not found any change in the gene expression of the mitochondrial respiratory chain factors *Sdha* (complex II) and *Cox4i1* (complex IV) or in the mitochondrial oxygen consumption rate between genotypes. Considering these findings, we suggest that the increased redox activity after CB_1_ receptor blockade (Arrabal et al., 2015) is likely produced in response to buffer a putative increase in reactive oxygen species. A buffered redox state under basal conditions could be one of the reasons for the lack of differences in mitochondrial respiration between genotypes, but can be challenged under an energy imbalance.

As the functional significance, the activation of CB_1_ receptors localized in different body tissues (brain, liver, skeletal muscle, pancreas, adipose tissue) participates in cellular and metabolic functions of the organism (Piomelli, 2003; Silvestri and Di Marzo, 2013; Iannotti et al., 2014; Mazier et al., 2015; Palomba et al., 2015) and regulates the mitochondrial biogenesis in non-neuronal peripheral tissues (Aquila et al., 2010; Tedesco et al., 2010). There are pieces of evidence indicating that mitochondria contain G proteins (Lyssand and Bajjalieh, 2007; Andreeva et al., 2008) and their effector signaling molecules such as soluble adenyl cyclase (Zippin et al., 2003), phosphodiesterase (Acin-Perez et al., 2011) and protein kinase A (PKA) (Ryu et al., 2005). Hence, cAMP generated in the mitochondria would lead to PKA activation and protein phosphorylation, thus regulating mitochondrial respiration and energy production (Chen et al., 2004; Helling et al., 2008; Acin-Perez et al., 2011). The oxidative phosphorylation carried out in mitochondria transforms the energy of the nutrients into ATP. The mitochondria regulate important physiological processes constantly adapting structure and functions to maintain the cellular metabolic homeostasis. In fact, mitochondria dysfunction is involved in neurodegenerative diseases obesity, insulin resistance and type 2 diabetes (Højlund et al., 2008; Bournat and Brown, 2010; Sivitz and Yorek, 2010; Dietrich et al., 2013; Schneeberger et al., 2013).

The skeletal muscle is the primary organ for nutrients and fatty acids oxidation, as well as glucose uptake. Insulin resistance is associated with a loss of muscular oxidative capacity in response to an increase of intake of fat-rich foods (Cavuoto et al., 2007a). The insulin resistance plays an important role in the pathogenesis of the metabolic syndrome and type 2 diabetes (Kelley et al., 2002; Kelley, 2005), while the blocking of CB_1_ receptors in the skeletal muscle has a direct effect on energy expenditure and oxidative metabolism (Ravinet Trillou et al., 2003; Liu et al., 2005; Arrabal et al., 2015). Skeletal muscle cells have been shown to contain CB_1_ and CB_2_ receptors as well as the endocannabinoid enzymatic machinery (DAGLα, DAGLβ, and MAGL) (Crespillo et al., 2011), and also the localization of CB_1_ receptors in striated muscle mitochondria was identified (Arrabal et al., 2015). These observations altogether support an endocannabinoid regulation of genes controlling the skeletal muscle metabolism (Crespillo et al., 2011). Therefore, it is presumable that the opposite effects of endocannabinoids and CB_1_ agonists reducing mitochondrial function in metabolically active tissues (Tedesco et al., 2010), and of CB_1_ antagonism (Tedesco et al., 2008), are through CB_1_ receptors in the mitochondria.

In conclusion, CB_1_ receptors are localized in the mitochondria of striated muscles in a similar proportion to brain tissue. Interestingly, most of the CB_1_ receptors in the gastrocnemius, the rectus abdominis and the myocardium are distributed in the mitochondria, in contrast to the brain where they are preferentially localized in neuronal membranes (Freund et al., 2003; Kano, 2014; Di Marzo et al., 2015). Furthermore, the activation of the mitochondrial CB_1_ receptors in striated muscle may participate in the regulation of the oxidative activity at the muscle mitochondria probably through the relevant enzymes implicated in the pyruvate metabolism, a main substrate for the Krebs cycle activity.

## Author contributions

Substantial contributions to the conception and design of the work, the acquisition, analysis, and interpretation of data: JMZ, SM, GB, AR, LR, SA, IE, IG, JS, FR, NP, GM, and PG. Drafting the work or revising it critically for important intellectual content: JMZ, SM, GB, AR, LR, SA, IE, IG, JS, FR, NP, GM, and PG. Final approval of the version to be published: JMZ, SM, GB, AR, LR, SA, IE, IG, JS, FR, NP, GM, and PG. Agreement to be accountable for all aspects of the work in ensuring that questions related to the accuracy or integrity of any part of the work are appropriately investigated and resolved: JMZ, SA, GB, AR, LR, IE, IG, JS, FR, NP, SM, GM, and PG.

## Funding

This work was supported by The Basque Government grant BCG IT764-13 (to PG); SAF2015-65034-R (MINECO/FEDER to PG); University of the Basque Country UPV/EHU UFI11/41 (to PG); Red de Trastornos Adictivos-Instituto de Salud Carlos III grant RD12/0028/0004 and RD16/0017/0012 (to PG); Instituto de Salud Carlos III, MINECO, UE-ERDF (CP12/03109 to JS); Red de Trastornos Adictivos (RD12/0028/0001 to FR); Consejería de Economía, Innovación y Ciencia, Junta de Andalucía, UE/ERDF (P-11-CVI-07637 to FR); Consejería de Salud, Junta de Andalucía (SAS111224 to FR); INSERM (to GM); EU–FP7 (PAINCAGE, HEALTH-603191, to GM); European Research Council (Endofood, ERC–2010–StG–260515); Fondation pour la Recherche Medicale (DRM20101220445 to GM); Human Frontiers Science Program (to GM); Region Aquitaine (to GM); Agence Nationale de la Recherche (ANR Blanc ANR-13-BSV4–0006-02 to GM). JS holds a “Miguel Servet” research contract from the National System of Health, ISCIII (grant number: CP12/03109).

### Conflict of interest statement

The authors declare that the research was conducted in the absence of any commercial or financial relationships that could be construed as a potential conflict of interest.

## Abbreviations

2-AG: 2-arachidonoylglycerol
ATP: adenosine triphosphate
BSA: bovine serum albumin
cAMP: cyclic adenosine monophosphate
CB_1_: cannabinoid type 1 receptor
*CB*_*1*_-KO: lack of cannabinoid type 1 receptor
CNS: central nervous system
DAGLα, DAGLβ: diacylglycerol lipase α, β
Δ^9^-THC: delta-9-tetrahydrocannabinol
MAGL: monoacylglycerol lipase
mtCB_1_: mitochondrial cannabinoid type 1 receptor
OCR: oxygen consumption ratio
OsO4: osmium tetroxide
PB: phosphate buffer
PBS: phosphate-buffered saline
PKA: protein kinase A
qRT-PCR: quantitative reverse transcription polymerase chain reaction
RCR: respiratory coupling ratio
RT: room temperature
TBS: Tris-HCl buffered saline
WT: wild type.
